# Dysregulated complement activation in COVID-19: the role of soluble complement inhibitors

**DOI:** 10.1093/cei/uxag064

**Published:** 2026-10-16

**Authors:** Stef van der Meulen, Haiyu Wang, Saskia Nugteren, Frederique Ponchel, Anna H E Roukens, Cilia R Pothast, Mirjam H M Heemskerk, Kyra A Gelderman, Jeroen C J Eikenboom, Bernard M van den Berg, Wendy Stam, Coert Margadant, Simone A Joosten, Leendert A Trouw, BEAT-COVID study group, M S Arbous, S Cannegieter, C M Cobbaert, A van der Does, J J M van Dongen, M C M Feltkamp, A Geluk, J J Goeman, M Giera, T Hankemeier, P S Hiemstra, C H Hokke, J J Janse, S P Jochems, M Kikkert, L Lamont, J Manniën, T H M Ottenhoff, M R del Prado, N Queralt Rosinach, M Roestenberg, M Roos, H H Smits, E J Snijder, F J T Staal, R Tsonaka, A Verhoeven, L G Visser, J J C de Vries, D J van Westerloo, J Wigbers, H J van der Wijk, R C van Wissen, M Wuhrer, M Yazdanbakhsh, M Zlei

**Affiliations:** Department of Immunology, Leiden University Medical Center, Leiden, The Netherlands; Department of Immunology, Leiden University Medical Center, Leiden, The Netherlands; Department of Immunology, Leiden University Medical Center, Leiden, The Netherlands; Leeds Institute of Rheumatic and Musculoskeletal Medicine, University of Leeds, Leeds, UK; Département de Rhumatologie, Université Jean Monnet Saint-Etienne, CHU Saint-Etienne, INSERM, Mines Saint-Etienne, SAINBIOSE U1059, Saint-Etienne, France; Leiden University Center for Infectious Diseases (LUCID), Leiden University Medical Center, Leiden, The Netherlands; Department of Hematology, Leiden University Medical Center, Leiden, The Netherlands; Department of Hematology, Leiden University Medical Center, Leiden, The Netherlands; Laboratory Medical Immunology, Department of Immunology, Erasmus Medical Center, Rotterdam, The Netherlands; Department of Internal Medicine, Division of Thrombosis and Hemostasis, Leiden University Medical Center, Leiden, The Netherlands; The Einthoven Laboratory for Vascular and Regenerative Medicine, Department of Internal Medicine, Nephrology, Leiden University Medical Center, Leiden, The Netherlands; The Einthoven Laboratory for Vascular and Regenerative Medicine, Department of Internal Medicine, Nephrology, Leiden University Medical Center, Leiden, The Netherlands; Institute of Biology, Gorlaeus Laboratory, Leiden University, Leiden, The Netherlands; Institute of Biology, Gorlaeus Laboratory, Leiden University, Leiden, The Netherlands; Leiden University Center for Infectious Diseases (LUCID), Leiden University Medical Center, Leiden, The Netherlands; Department of Immunology, Leiden University Medical Center, Leiden, The Netherlands

**Keywords:** COVID-19, complement activation, soluble complement inhibitors

## Abstract

In viral infections, complement activation can contribute to viral clearance, but excessive activation, as observed in severe coronavirus disease 2019 (COVID-19), may drive immunopathology. While much is known about complement-activating factors in COVID-19, insight into the role of endogenous complement inhibitors remains limited. We performed a longitudinal analysis within the BEAT-COVID cohort, comprising hospitalized patients with severe acute respiratory syndrome coronavirus 2 infection admitted to the intensive care unit (ICU) or ward, and age-matched healthy controls. Serum levels of the complement activation marker soluble membrane attack complex (sC5b-9) and soluble inhibitors C1-inhibitor, C4b-binding protein (C4BP), factor H, and factor I were measured by enzyme-linked immunosorbent assay. *In vitro* endothelial complement sensitivity was assessed by flow cytometry. Soluble inhibitors were also measured in a cohort of patients with long COVID. Patients admitted to the ICU showed elevated sC5b-9 levels compared to patients admitted to the ward and controls, correlating with disease severity, length of hospitalization, and need for mechanical ventilation, but not mortality. Unexpectedly, levels of all soluble complement inhibitors were elevated, particularly in patients admitted to the ICU. Factor H levels positively correlated with sC5b-9. Endothelial cells pre-incubated with serum from patients admitted to the ICU became more susceptible to complement attack. In patients with long COVID, factor H and C4b-binding protein levels remained elevated compared to pre-pandemic healthy controls. Severe COVID-19 is characterized by extensive complement activation despite elevated circulating inhibitor levels, suggesting impaired local complement regulation at the cell surface, potentially amplified by overwhelming systemic activation, rather than inhibitor deficiency.

## Teaser

In viral infections, complement activation can contribute to viral clearance, but excessive activation, as observed in severe COVID-19, may drive immunopathology. While much is known about complement-activating factors in COVID-19, insight into the role of endogenous complement inhibitors remains limited.

## Graphical Abstract

**Graphical Abstract:**
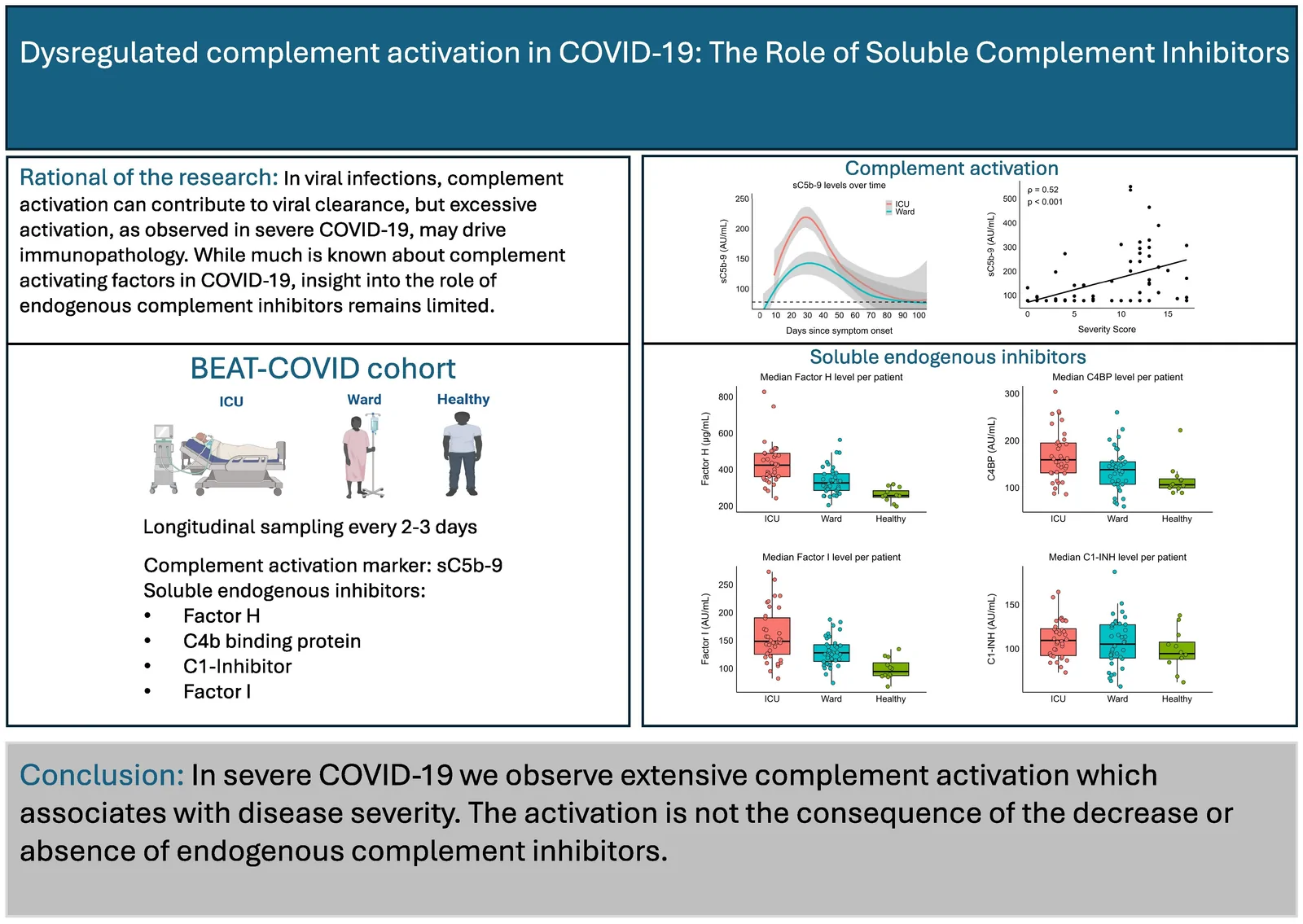

## Introduction

The global outbreak of coronavirus disease 2019 (COVID-19), caused by the severe acute respiratory syndrome coronavirus 2 (SARS-CoV-2), has posed major challenges to public health systems worldwide [1]. COVID-19 displays a broad clinical spectrum, ranging from asymptomatic infection to severe pneumonia, acute respiratory distress syndrome (ARDS), multi-organ failure, and death [2]. Severe disease results from complex interactions between viral pathogenicity, host immune responses, and systemic inflammation [3].

An important component of the immune response in COVID-19 is the complement system [4]. The complement system is a central part of innate immunity and comprises more than 30 serum proteins that recognize and eliminate pathogens while discriminating self from non-self in a highly regulated manner [5]. Emerging evidence indicates that excessive complement activation contributes significantly to the pathophysiology of severe COVID-19 [6–9]. SARS-CoV-2 can directly activate complement via its spike and nucleocapsid proteins, which are recognized by components of the classical, lectin, and alternative pathways [10–12]. This activation promotes inflammation, endothelial injury, and thromboinflammation, which are key features observed in severe COVID-19 cases [13, 14].

Under steady-state conditions, complement activity is controlled by endogenous inhibitors, while excessive activation may result from increased activation or impaired inhibition [15]. Immune complexes or pathogen surfaces can enhance complement activation, as observed in autoimmune diseases and infections [16–18], whereas defective inhibition occurs in genetic deficiencies or in the presence of autoantibodies targeting complement inhibitors [19, 20]. Regulation of the complement system on the cell surface, but especially in the fluid-phase, is crucial to prevent excessive activation and resultant tissue damage.

Several soluble endogenous complement inhibitors, including C1-inhibitor (C1-INH), C4b-binding protein (C4BP), factor H, and factor I, play vital roles in maintaining this balance [15]. C1-INH regulates the classical and lectin pathways by inhibiting the complement-activating enzymes [C1r, C1s, mannose-binding lectin-associated serine protease-1 (MASP-1), and MASP-2] [21]. C4BP functions as an inhibitor by binding to C4b. It accelerates the decay of the C3-convertase and serves as a co-factor for factor I that will cleave C4b into an inactive fragment [22]. Although less efficiently, C4BP will also facilitate factor I mediated degradation of C3b [23]. Factor H is the key inhibitor of the alternative pathway. It ensures that complement activation is appropriately directed toward pathogens and not host tissues, by promoting the decay of C3-convertase and acting as a co-factor for factor I in the inactivation of C3b. Factor I works in conjunction with factor H and C4BP to cleave and inactivate C3b and C4b, thereby preventing the formation of C3 and C5 convertases. Reduced levels of complement inhibitors have also been observed in autoimmune diseases with pronounced complement activation, such as systemic lupus erythematosus [24].

Complement activation results from the balance between activation and inhibition. The complement activation observed in COVID-19 could therefore be the consequence of overactivation or of insufficient complement regulation. In this study, we therefore examined the levels of soluble complement inhibitors, complement activation, and disease progression in patients with COVID-19 utilizing a unique cohort with longitudinal data points. This cohort allowed us to investigate the dynamic interactions between complement activation and immune responses over time, providing valuable insights into how complement may contribute to immunopathology and coagulopathy.

## Methods and materials

### Study design and ethics

The BEAT-COVID cohort was collected at the Leiden University Medical Center (LUMC), Leiden, The Netherlands. Individuals diagnosed with polymerase chain reaction-positive COVID-19 who required hospital admission between April 2020 and March 2021 were invited to participate [25–28]. Ethical approval for the study protocol was provided by LUMC Medical Ethics Committee (protocol NL73740.058.20). The study was registered at the International Clinical Trials Registry Platform No. NL8589 (https://trialsearch.who.int/Trial2.aspx?TrialID=NL8589). The study complied with the latest version of the Declaration of Helsinki. The principal investigator had access to information to identify individual patients. Details of the cohort and severity of coronavirus disease assessment (SCODA) severity scores [29] are available in the online supplementary material.

In addition, an independent cohort of individuals with persistent symptoms for >12 weeks consistent with a diagnosis of long COVID, together with a group of fully recovered post-COVID participants, was recruited in the United Kingdom. Ethical approval for this cohort was obtained from the UK Research Ethics Committee (REC-21/EM0112). Details of the long COVID cohort have been published previously [30].

### Sample collection and storage

All samples of the BEAT-COVID cohort were collected following a strict standardized protocol. In brief, blood samples were collected by venipuncture into an 8.5 ml serum separator tube Vacutainer tube (Becton Dickinson, VWR). Upon clotting, samples were centrifuged (10 min, 3000 rpm) within 1 h after sample collection by the central clinical chemistry laboratory and aliquoted for research purposes. Samples were immediately stored at −80°C to ensure optimal preservation of sample quality.

All samples in the BEAT-COVID study from intensive care unit (ICU) patients, ward patients, and healthy controls were collected and processed using an identical standardized protocol, so that any matrix-related contribution to baseline complement activation affected all groups equally. Following thawing, each aliquot was immediately diluted 1:10 in phosphate-buffered saline (PBS) containing 20 mM ethylenediaminetetraacetic acid (EDTA) to prevent further ex vivo complement activation. sC5b-9 was measured directly after the initial thaw, whereas soluble complement regulators were measured on aliquots stored at −20°C and analyzed within 1 month. All BEAT-COVID aliquots used for the enzyme-linked immunosorbent assays (ELISAs) described here therefore underwent only a single freeze–thaw cycle prior to measurement.

Blood from the Leeds participants was initially collected as 9 ml of clotted whole blood, from which serum was separated by centrifugation within 1 h of collection and stored at −80°C. However, in contrast to the BEAT-COVID samples, the long COVID serum samples from Leeds were not diluted in EDTA buffer upon initial thawing, and aliquots had undergone multiple freeze–thaw cycles prior to analysis at our laboratory. These pre-analytical differences rendered reliable quantification of sC5b-9 not feasible in the long COVID cohort, and measurements in this cohort were therefore restricted to the soluble complement inhibitors, which are more robust to repeated freeze–thaw cycles.

### Enzyme-linked immunosorbent assays

Four in-house sandwich ELISAs were used to quantify serum complement inhibitors, and an additional in-house ELISA quantified soluble C5b-9 (sC5b-9). MaxiSorp plates (Thermo Scientific, Nunc, 430341) were coated overnight at room temperature with capture antibodies (50 µl/well) diluted in coating buffer (0.1 M Na_2_CO_3_/NaHCO_3_, pH 9.6): rabbit anti-human C1-INH (Thermo Fisher, PA5-81015; 0.33 µg/ml), rabbit anti-human C4BP (kindly provided by Prof. dr. Anna M. Blom, Malmö, Sweden; 2.5 µg/ml), mouse anti-human factor H (Sanquin, clone aFH16; 0.5 µg/ml), goat anti-human factor I (CompTech, A238; 0.1 µg/ml), or mouse anti-human C5b-9 (Dako, clone aE11; 15 µg/ml).

Between all steps, plates were washed three times with PBS/0.05% Tween-20, blocked with PBS/1% bovine serum albumin (BSA) (100 µl/well, 1 h, 37°C), and incubated with serum diluted in PBS/0.05% Tween-20/1% BSA (PTB buffer) for 1 h at 37°C (50 µl/well). Serum dilutions were 1:10 000 (C1-INH), 1:4000 (C4BP α-chain), 1:1000 (C4BP β-chain), 1:4000 (factor H), 1:150 (factor I), and 1:50 (C5b-9).

Detection antibodies were goat anti-human C1-INH (CompTech, A240), mouse anti-human C4BP α-chain (Quidel, A215), mouse anti-human C4BP β-chain (clone 2B, kindly provided by Prof. dr. Anna M. Blom), biotinylated mouse anti-human factor H (OX-23), mouse anti-human factor I (MRCOX-21), and goat anti-human C5 (Quidel, A306). Secondary antibodies included Horseradisch Peroxidase (HRP)-labeled rabbit anti-goat IgG (H + L) (Invitrogen, A16142) for C1-INH and C5b-9, HRP-labeled goat anti-mouse Ig (Dako, P0447) for C4BP and factor I, and HRP-labeled streptavidin (Thermo Scientific, 21126) for factor H. All antibodies were diluted in PTB buffer.

Plates were developed with 2,2′-azino-bis(3-ethylbenzothiazoline-6-sulfonic acid) (ABTS) (Merck, A1888-5G) containing 1:2000 H_2_O_2_ (Merck, 107209), and absorbance was measured at 415 nm using a Bio-Rad iMark microplate reader. sC5b-9 was calibrated against maximally activated pooled serum from healthy controls and expressed as arbitrary units (AU)/ml. C1-INH, C4BP, and factor I were quantified using serial dilutions of pooled normal human serum (NHS) (AU/ml), while factor H was expressed in µg/ml using calibrated reference serum (Sanquin).

### ELISA for IgG reactivity against complement regulators

IgG antibodies against factor H, Factor I, C4BP and C1-INH were measured by ELISA in the serum samples from the BEAT-COVID cohort. Nunc MaxiSorp plates were coated with 3 µg/ml purified factor H (CompTech, A137), factor I (CompTech, A138), C4BP (CompTech, A109), C1-INH (CompTech, A140), and BSA (Sigma-Aldrich, A7906-500G) in coating buffer (0.1 M Na_2_CO_3_/NaHCO_3_, pH 9.6) in 50 µl/well overnight at 4°C. BSA served as a negative control antigen to quantify non-specific IgG binding for each individual sample. Plates were washed with PBS/0.05% Tween-20 and blocked with PBS/1% BSA in 100 µl/well, 1 h, 37°C. Serum samples were diluted 1:50 in PBS/0.05% Tween-20/1% BSA (PTB buffer) for 1 h at 37°C in 50 µl/well. Bound IgG was detected with 1:5000 rabbit anti-human IgG-HRP (Dako, P0214) in PTB for 1 h at 37°C in 50 µl/well. Plates were developed with ABTS (Merck, A1888-5G) containing 1:2000 H_2_O_2_ (Merck, 107209), and absorbance was measured at 415 nm using a Bio-Rad iMark microplate reader. Results are expressed in AU relative to a standard of human IgG included on every plate.

BSA reactivity was used as quality control and was applied per patient: patients with a BSA signal 1.25× the lower limit of the assay in any sample were excluded entirely. This removed three ICU patients (44%/60%/67% of their samples were BSA-reactive), leaving 33 ICU patients, 38 ward patients and 12 healthy controls. For the remaining samples, all absorbance values were calculated into AU/ml. Next, the BSA signal was subtracted from the antigen-specific signal and a sample was scored positive when this corrected value exceeded zero. A cut-off based on the healthy control distribution was not feasible, as all healthy controls were at the lower limit of the assay for anti-factor H, anti-C4BP, and anti-factor I, and the only anti-C1-INH variation came from a single donor. Positivity was therefore defined relative to each sample’s own BSA control, and a patient was scored positive for a given autoantibody only when at least two of their samples were positive.

### *In vitro* endothelial complement opsonization assay

Human umbilical vein endothelial cells (Lonza, C2519A) were cultured in endothelial cell growth medium 2 (EGM-2) medium (PromoCell, C-22111) in T75 flasks (CELLSTAR®, 658175) coated with 0.1% gelatin (Merck, 1.04078) at 37°C to ∼90% confluency. For experiments, 12-well plates (Corning, 3512) were coated with 0.1% gelatin, seeded with 1 × 10^5^ cells/well, and incubated for 24 h.

Cells were washed twice with PBS and incubated overnight at 37°C with 10% pooled ICU or ward serum, or 10% fetal calf serum as control, diluted in EGM-2. After washing, cells were incubated for 1 h at 37°C with 40% complement-active or heat-inactivated serum (56°C, 30 min) diluted in Rosewell Park Memorial Institute (RPMI) 1640 medium (Gibco™, 22409015), followed by two PBS washes.

Cells were detached using Trypsin (Sigma-Aldrich, 59418C) and transferred to 96-well plates. Viability was assessed using Zombie Aqua™ (1:1000, BioLegend, 423101; 20 min, room temperature, dark). Cells were stained for 30 min at 4°C with rabbit anti-human C3c- fluoresceïne-isothiocyanaat (10 µg/ml, Agilent, F0201) and mouse anti-human C5b-9 (1:400, clone aE11, Agilent, M0777). After washing, C5b-9 was detected using donkey anti-mouse AF647 (1:200, Invitrogen, 31571). Samples were analyzed on a Cytek® Aurora flow cytometer (Cytek Biosciences, Fremont, CA, USA).

### Statistics

Statistical analyses were performed in R (version 4.4.0; R Foundation for Statistical Computing). Data are presented as median (interquartile range). Two-group comparisons used the Mann–Whitney U test; comparisons of more than two groups used the Kruskal–Wallis test with Dunn’s *post hoc* test and Bonferroni correction. Correlations were assessed using Spearman’s rank correlation coefficient. A *P*-value <0.05 was considered statistically significant. Analyses used the Simple Fischeries Stock Assessment Methods, dunn.test, Hmisc, tidyverse, and ggplot2 packages.

## Results

### Demographics

The analysis included 86 participants from the BEAT-COVID study who were longitudinally followed at the LUMC between March 2020 and February 2021. The full BEAT-COVID cohort comprised 192 patients; the present analysis was restricted to the first 86 individuals for whom complete longitudinal sample sets were available at the time of study initiation. Demographic and clinical characteristics are summarized in Table 1.

**Table 1:** Demographic and clinical characteristics of the BEAT-COVID cohort.

	ICU (*n* = 36)	Ward (*n* = 38)	Healthy control (*n* = 12)
Male	26 (72%)	30 (79%)	8 (67%)
Age in years ± SD	64 ± 11	62 ± 15	64 ± 4
BMI ± SD	27.9 ± 3	29.1 ± 8	
*Comorbidities*			
Chronic cardiac disease (not hypertension)	9 (25%)	11 (29%)	
Hypertension	11 (31%)	10 (26%)	
Chronic pulmonary disease (not asthma)	1 (3%)	1(3%)	
Asthma (physician diagnosed)	9 (25%)	4 (11%)	
Chronic kidney disease	3 (8%)	3(8%)	
Chronic liver disease	1 (3%)	1 (3%)	
Chronic neurological disorder	4 (11%)	3(8%)	
AIDS/HIV	0 (0%)	0 (0%)	
Diabetes	13 (36%)	11 (29%)	
Tuberculosis	0 (0%)	1 (3%)	
Prior thromboembolic event	6 (17%)	1 (3%)	
Coagulopathy	1 (3%)	1 (3%)	
Organ transplant	1 (3%)	3 (8%)	
*Outcome*			
Average length of hospitalization in days ± SD	34 ± 19	9 ± 6	
Invasive ventilation	18 (50%)	1 (3%)	
*In-hospital mortality*	12 (33%)	3 (8%)	
Thromboembolic event (e.g. DVT and PE)	5	0	
ARDS	9	2	
Bleeding	1	0	
Acute renal injury	7	1	

Demographic and clinical characteristics of the 74 patients included in the BEAT-COVID study and the 12 healthy controls. Patients were categorized as ICU if they were admitted to the intensive care unit at any point during hospitalization; those not admitted to the ICU were classified as ward. Healthy controls (*n* = 12) were ≥60 years of age and selected to match the demographic profile of the patient cohort. The table summarizes baseline characteristics, comorbidities, and clinical outcomes, including length of hospitalization, need for invasive ventilation, and in-hospital mortality. Data are presented as number (percentage) or mean ± standard deviation (SD).

Abbreviations: AIDS, acquired immunodeficiency syndrome; ARDS, acute respiratory distress syndrome; BMI, body mass index; DVT, deep vein thrombosis; HIV, human immunodeficiency virus; ICU, intensive care unit; PE, pulmonary embolism; SD, standard deviation.

Of the 86 participants, 74 were diagnosed with COVID-19, of whom 36 were admitted to the ICU at any point during hospitalization and 38 were hospitalized on a regular ward; 15 COVID-19 patients died during hospitalization. In parallel, 12 healthy donors were enrolled using the same workflow. Serum samples were collected longitudinally every 2–3 days during hospitalization and once at 6 weeks post-discharge.

### Complement activation is associated with disease severity and the need for mechanical ventilation

Soluble C5b-9 (sC5b-9) levels were measured in serum samples at each longitudinal time point as a marker of complement activation and levels are displayed for all participants (Fig. 1a, *X*-axis) at multiple time points (*Y*-axis). ICU patients showed markedly higher sC5b-9 levels than ward patients, while healthy controls showed no evidence of complement activation.

**Figure 1:**
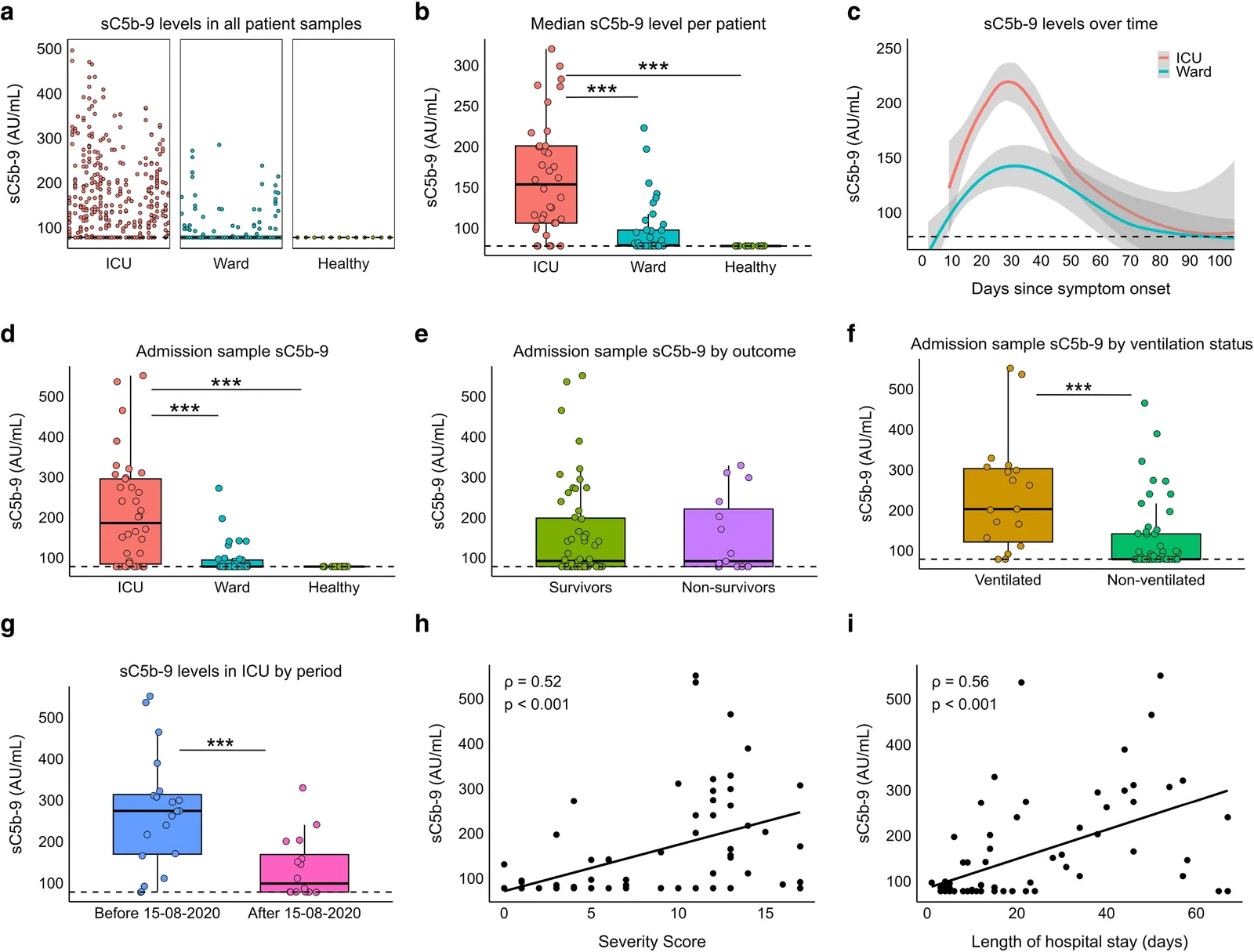
sC5b-9 levels correlate with disease severity in patients with COVID-19. (a) Comparison of sC5b-9 levels among ICU patients, ward patients, and healthy controls in all samples of the BEAT-COVID cohort. Each point on the *x*-axis represents an individual patient; corresponding coordinates on the *y*-axis indicate the values of all samples collected over time from that patient. (b) Median sC5b-9 levels per patient across the entire hospitalization were calculated and compared between groups using the Kruskal–Wallis test with Dunn’s *post hoc* test. *P < 0.05; ***P < 0.001. (c) Longitudinal analysis of sC5b-9 levels normalized to days since symptom onset. (d) sC5b-9 levels in the first sample collected after admission. ICU patients showed significantly elevated sC5b-9 levels compared to ward patients and healthy controls (*P* < 0.001), while ward patients had similar levels to controls. Statistical significance was determined using the non-parametric Kruskal–Wallis test followed by Dunn’s *post hoc* test. ***P < 0.001. (e) Comparison of sC5b-9 levels at admission between non-survivors and survivors, no differences between groups. (f) Comparison of sC5b-9 levels at admission between patients who did and did not require mechanical ventilation. Statistical comparison was performed using the Mann–Whitney U non-parametric test. ***P < 0.001. (g) Comparison of admission samples from patients in the first wave (before 15 August 2020) and the second wave (after 15 August 2020) of the SARS-CoV-2 virus in The Netherlands (*P* < 0.001). Statistical comparison was performed using the Mann–Whitney U non-parametric test. ***P < 0.001. (h) Correlation between sC5b-9 levels at admission and severity score, showing that the degree of complement activation is associated with higher disease severity (*ρ* = 0.52, *P* < 0.001). Statistical analysis was performed using Spearman correlation. (i) Correlation between sC5b-9 levels at admission and length of hospitalization, showing that the degree of complement activation is associated with longer hospital stay (*ρ* = 0.56, *P* < 0.001). Statistical analysis was performed using Spearman correlation. LLOQ = lower limit of quantification, indicated by the black dotted horizontal line.

As direct longitudinal group-wise comparisons were not feasible, median sC5b-9 levels per patient across hospitalization were calculated and compared between groups. ICU patients had significantly higher median sC5b-9 levels than ward patients and healthy controls (Fig. 1b, both *P* < 0.001), whereas no difference was observed between ward patients and controls (*P* = 0.11).

Longitudinal analysis normalized to days since symptom onset demonstrated consistently higher sC5b-9 levels in ICU patients compared to ward patients, with both groups peaking approximately 30 days after symptom onset, followed by a gradual decline toward the lower detection limit (Fig. 1c).

Analysis of the first serum sample obtained after admission revealed significantly higher sC5b-9 levels in ICU patients compared with ward patients and healthy controls (Fig. 1d, both *P* < 0.001), while ward patients again showed levels comparable to controls. At admission, sC5b-9 levels did not differ between survivors and non-survivors (Fig. 1e, *P* = 0.77). In contrast, sC5b-9 levels at admission were significantly higher in patients who required invasive mechanical ventilation than in those who did not (Fig. 1f, *P* < 0.001). Patients in the high sC5b-9 group had an increased risk of requiring mechanical ventilation (odds ratio 8.1, 95% confidence interval 2.3–39.8; *P* = 0.001).

Among ICU patients, sC5b-9 levels were significantly higher during the first COVID-19 wave than during the second wave in the Netherlands (Fig. 1g, *P* < 0.001). Across all hospitalized patients, sC5b-9 levels at admission correlated positively with disease severity scores [29] (Fig. 1h, *ρ* = 0.52, *P* < 0.001) and length of hospitalization (Fig. 1i, *ρ* = 0.56, *P* < 0.001), indicating that elevated complement activation is associated with increased disease severity.

### Elevated levels of complement inhibitors in the classical and lectin pathway

We next measured serum concentrations of C1-INH and C4BP in all available samples from each patient and displayed as before (Fig. 2a and b). At admission, C1-INH levels were elevated in patients admitted to both the ICU and the ward compared with healthy controls (Fig. 2c, *P* = 0.02 and *P* = 0.04), with no difference between patient groups. In contrast, C4BP levels did not differ between the three groups at admission (Fig. 2d).

**Figure 2:**
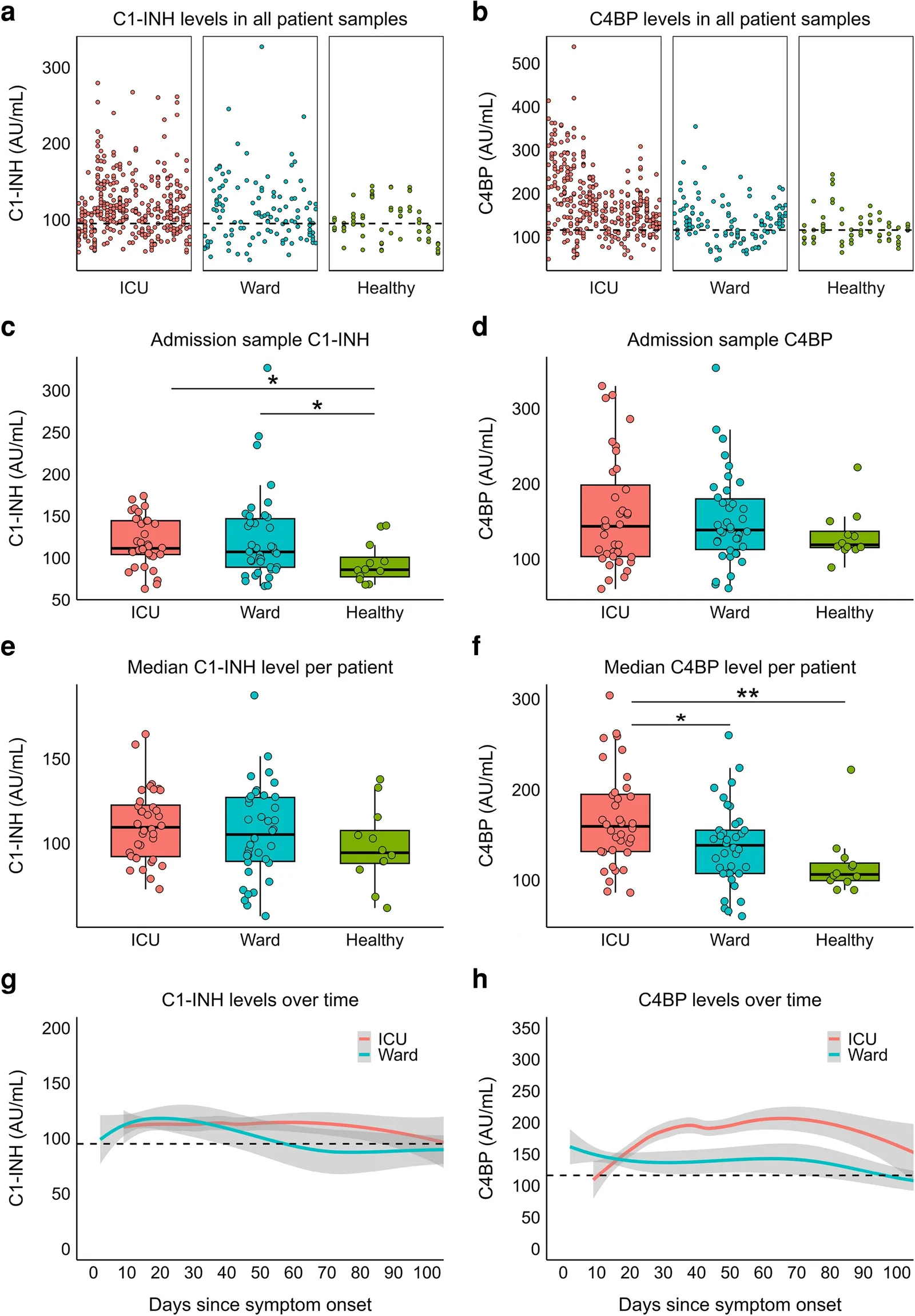
Serum levels of C1-INH and C4BP in COVID-19 patients. Levels of C1-INH (a) and C4BP (b) in all samples from ICU patients, ward patients, and healthy controls. Each point on the *x*-axis represents an individual patient; corresponding coordinates on the *y*-axis indicate the values of all samples collected over time from that patient. (c) Comparison of C1-INH levels in admission samples. Both ICU and ward patients showed higher levels than healthy controls (*P* = 0.02 and *P* = 0.04, respectively), with no difference between ICU and ward patients. Statistical comparison was performed using the non-parametric Kruskal–Wallis test, followed by Dunn’s *post hoc* test. *P < 0.05. (d) Comparison of C4BP levels in admission samples showing no significant differences between groups. (e) Comparison of median C1-INH levels per patient across the entire hospitalization revealed no significant differences between groups. (f) Comparison of median C4BP levels per patient across the entire hospitalization showed significantly higher levels in ICU patients compared with healthy controls (*P* = 0.019) and ward patients (*P* = 0.04), whereas no difference was observed between ward patients and healthy controls. Statistical comparisons were performed using the non-parametric Kruskal–Wallis test followed by Dunn’s *post hoc* test. *P < 0.05; **P < 0.01. (g) Longitudinal analysis of C1-INH levels normalized to days since symptom onset showing stable levels over time across all groups. (h) Longitudinal analysis of C4BP levels normalized to days since symptom onset, showing an increase in the levels of C4BP in the first 40 days in the ICU group. Dotted line indicates the median value of the healthy controls.

When summarized as median levels per patient over time across groups, C1-INH remained comparable between groups (Fig. 2e), whereas C4BP showed a distinct pattern: patients admitted to the ICU had higher median C4BP levels than both healthy controls (Fig. 2f, *P* = 0.019) and ward patients (*P* = 0.04), while ward patients were comparable to controls. Longitudinal analysis normalized to days since symptom onset showed stable C1-INH levels across groups (Fig. 2g), whereas C4BP levels diverged over time, with an increase in ICU patients peaking between days 30 and 70, levels in ward patients remained close to healthy control medians throughout hospitalization (Fig. 2h).

### Increased levels of inhibitors of the alternative pathway

Despite pronounced complement activation in ICU patients, levels of the soluble alternative pathway inhibitors factor H and factor I were markedly increased (Fig. 3a and b). In admission samples, factor H concentrations were higher in ICU patients than in ward patients and healthy controls (Fig. 3c, *P* = 0.003 and *P* < 0.001), with ward patients also exceeding controls (*P* = 0.04). Factor I showed a similar pattern, with elevated levels in ICU and ward patients compared with healthy controls (Fig. 3d, *P* < 0.001 and *P* = 0.002), but no difference between ICU and ward patients.

**Figure 3:**
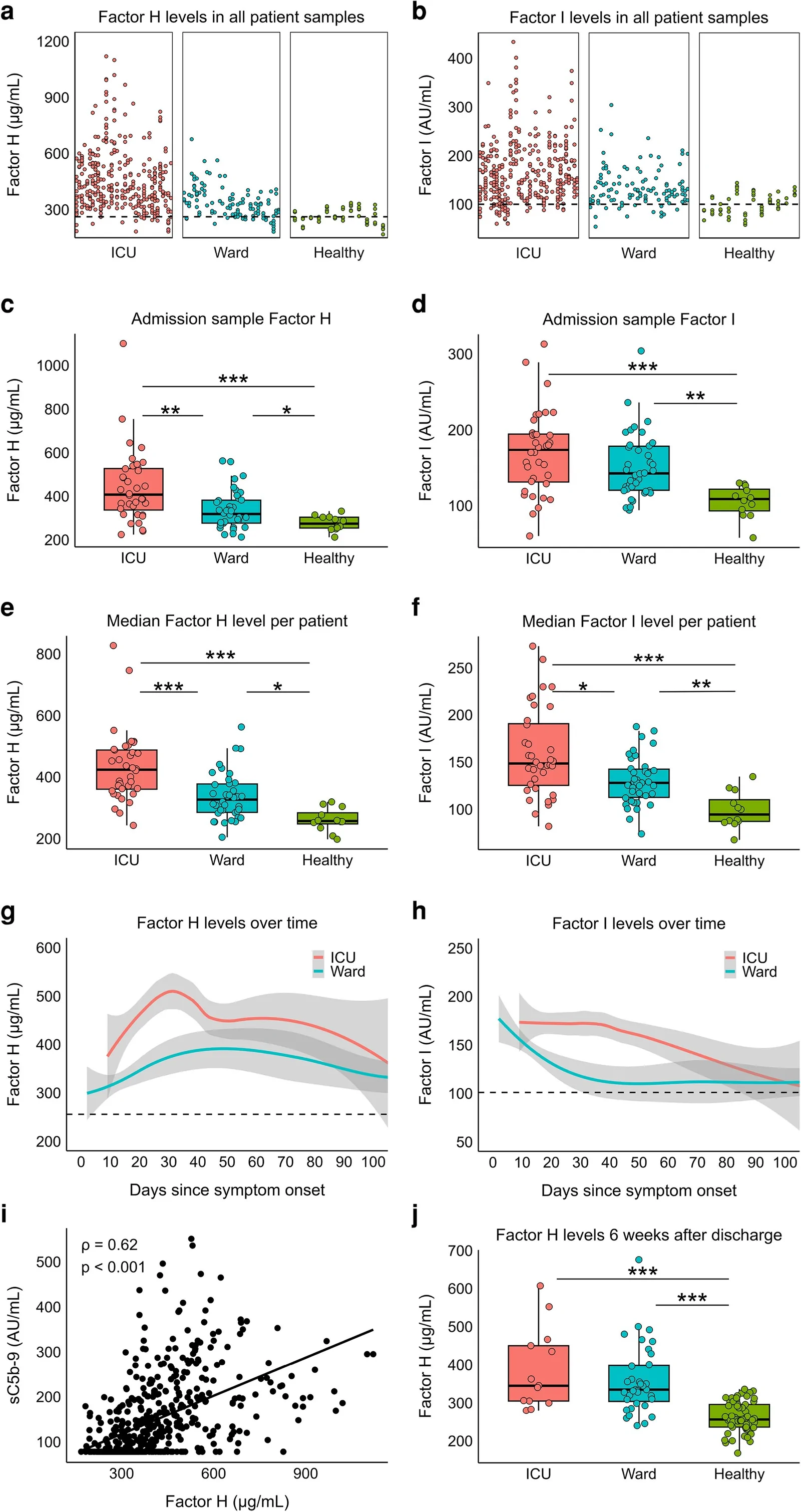
Levels of soluble complement inhibitors factor H and factor I in COVID-19 patients. Levels of factor H (a) and factor I (b) in all samples from ICU patients, ward patients, and healthy controls. Each point on the *x*-axis represents an individual patient; corresponding coordinates on the *y*-axis indicate the values of all samples collected over time from that patient. (c) Comparison of factor H levels in admission samples showing higher levels in ICU patients compared to ward patients (*P* = 0.003) and healthy controls (*P* < 0.001), and higher levels in ward patients compared to healthy controls (*P* = 0.04). (d) Comparison of factor I levels in admission samples showing higher levels in ICU patients (*P* < 0.001) and ward patients (*P* = 0.002) compared to healthy controls, with no significant difference between ICU and ward patients. Statistical comparison for the admission samples was performed using the non-parametric Kruskal–Wallis test, followed by Dunn’s *post hoc* test. *P < 0.05; **P < 0.01; ***P < 0.001. (e) Comparison of median factor H levels per patient across the entire hospitalization showed significantly higher levels in ICU patients compared with both ward patients and healthy controls (*P* < 0.001 for both comparisons). Median factor H levels were also significantly higher in ward patients compared with healthy controls (*P* = 0.019). (f) Comparison of median factor I levels per patient across the entire hospitalization revealed the highest levels in ICU patients compared with both ward patients (*P* = 0.027) and healthy controls (*P* < 0.001). In addition, median factor I levels were significantly higher in ward patients compared with healthy controls (*P* = 0.007). Statistical comparisons were performed using the non-parametric Kruskal–Wallis test followed by Dunn’s *post hoc* test. *P < 0.05; **P < 0.01; ***P < 0.001. (g) Longitudinal analysis of factor H levels normalized to days since symptom onset. (h) Longitudinal analysis of factor I levels normalized to days since symptom onset. (i) Positive correlation between sC5b-9 levels and factor H (*ρ* = 0.62, *P* < 0.001), statistical analysis was performed using Spearman correlation. (j) Factor H levels remain elevated even 6 weeks post-discharge in both ICU and ward patients compared to the levels of healthy controls (both *P* < 0.001). Statistical comparison for the 6 weeks post-discharge was performed using the non-parametric Kruskal–Wallis test, followed by Dunn’s *post hoc* test. ***P < 0.001.

Using median levels per patient over time, factor H was highest in ICU patients and differed significantly from ward patients and healthy controls (Fig. 3e, both *P* < 0.001), while ward patients still showed higher levels than controls (*P* = 0.019). Median factor I levels were increased in ICU patients compared with healthy controls (Fig. 3f, *P* < 0.001) and ward patients (*P* = 0.027), and were also elevated in ward patients relative to controls (*P* = 0.007).

Longitudinal analysis revealed distinct kinetics. Factor H increased over time in both groups, reaching a peak around 30 days after symptom onset. Levels in both groups remained above healthy control medians (Fig. 3g). In contrast, factor I levels were initially elevated in both groups but normalized within 30 days in ward patients, whereas ICU patients maintained elevated levels for up to 40 days before a slow gradual decline up to 80 days (Fig. 3h). Notably, factor H levels correlated positively with sC5b-9 (*ρ* = 0.62, *P* < 0.001; Fig. 3i), contrary to the typically inverse relationship reported under other conditions [24]. Finally, factor H levels remained elevated in both ICU and ward patients 6 weeks post-discharge compared with healthy controls (both *P* < 0.001; Fig. 3j).

Since autoantibodies against complement regulators have been described in inflammatory conditions and could affect inhibitor levels or function, we screened all available samples for IgG reactivity against factor H, factor I, C4BP, and C1-INH by ELISA, using BSA as a control antigen. Patients with BSA reactivity were excluded, leaving 33 ICU patients, 38 ward patients, and 12 healthy controls for analysis. Autoantibody positivity was infrequent in all groups (online supplementary material, Fig. S1); anti-factor H (ICU 2/33, ward 4/38, healthy 0/12), anti-C4BP (6/33, 0/38, 0/12), anti-factor I (1/33, 0/38, 0/12), and anti-C1-INH (3/33, 2/38, 1/12). Fourteen patients were positive for at least one inhibitor, with a comparable overall frequency in ICU and ward patients (7/33 vs 6/38, *P* = 0.76, Fisher’s exact test). These data indicate that the elevated concentrations of soluble complement inhibitors observed in COVID-19 are unlikely to be driven by an autoantibody response.

### Enhanced complement activation on *in vitro* endothelial cells exposed to COVID-19 patient serum

Previous work from the BEAT-COVID consortium demonstrated that endothelial glycocalyx degradation in COVID-19 was associated with a pro-coagulant phenotype [31]. Since peak complement activation coincided with elevated inhibitor levels, we hypothesized that excessive activation could be the result of impaired inhibitor binding and cellular protection rather than inhibitor deficiency. Plasma von Willebrand factor (VWF), released upon endothelial activation or injury, was used as a marker of endothelial perturbation. Consistent with earlier reports [32, 33], ICU patients had higher VWF levels than patients admitted to the ward and healthy controls (Fig. 4a, both *P* < 0.001), while ward patients also differed from controls (*P* = 0.002). VWF levels correlated positively with complement activation across all patients with SARS-CoV-2 infection (Fig. 4b, *ρ* = 0.48, *P* < 0.001). These findings suggest that serum from severely ill patients with COVID-19 promotes endothelial susceptibility to complement activation.

**Figure 4:**
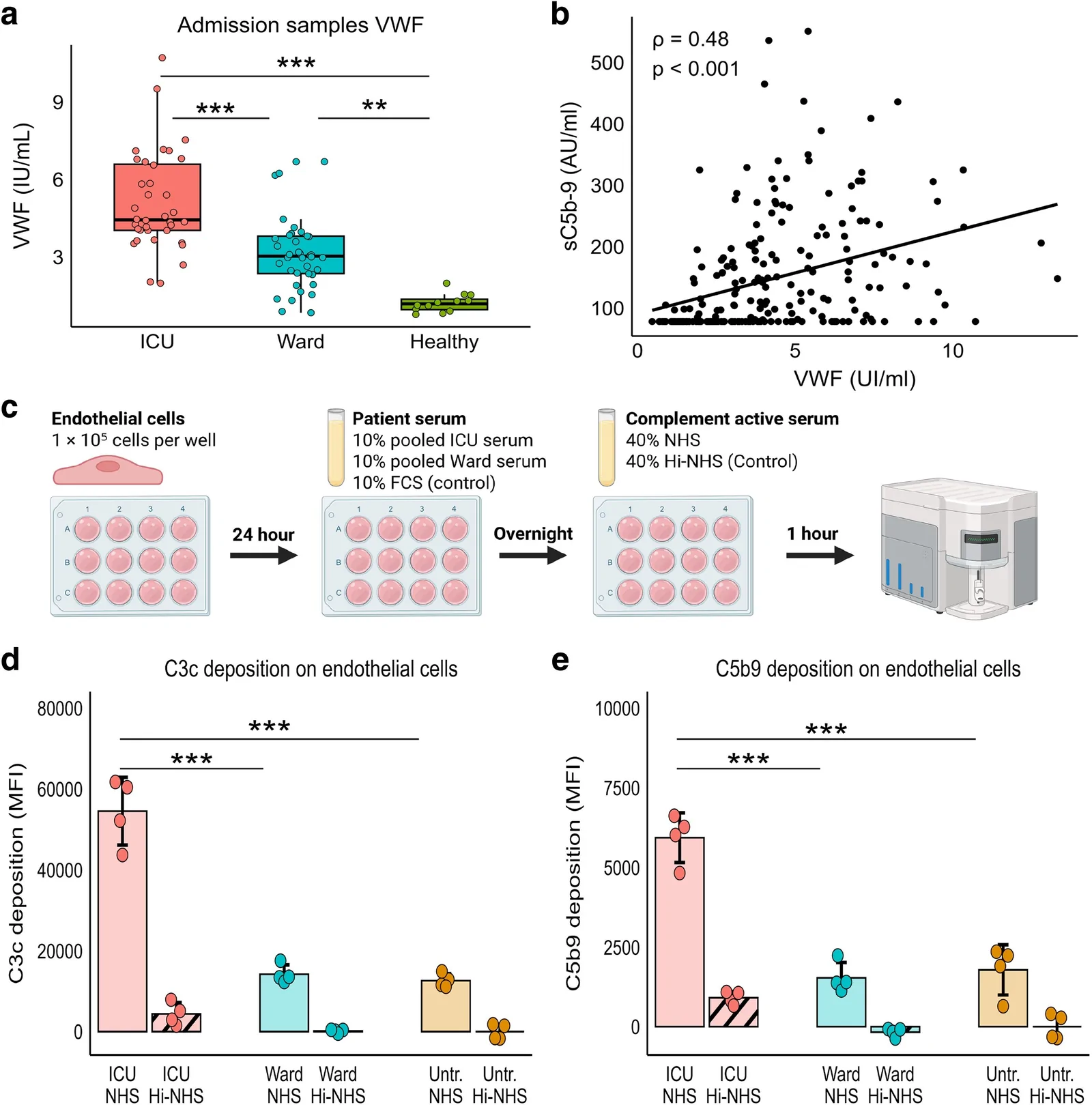
Increased cellular susceptibility to complement activation in endothelial cells. (a) Circulating VWF levels in ICU patients, ward patients, and healthy controls. ICU patients showed significantly higher VWF than ward patients and healthy controls (*P* < 0.001), and ward patients had higher VWF than controls (*P* = 0.002). Statistical significance was determined using the non-parametric Kruskal–Wallis test, followed by Dunn’s *post hoc* test. *P < 0.05; **P < 0.01; ***P < 0.001. (b) Correlation between VWF and serum sC5b-9 levels across all patients (*ρ* = 0.48, *P* < 0.001). Statistical analysis was performed using Spearman correlation. (c) Assay schematic for complement activation on endothelial cells. Created in BioRender https://BioRender.com/nvcn0pj (d and e) Endothelial cells were pre-incubated overnight with pooled ICU serum, pooled ward serum, or no-serum control, washed, followed by exposure to fresh complement-active serum (NHS) or heat-inactivated normal human serum. Flow cytometry analysis showed increased C3c opsonization and C5b-9 deposition after pre-incubation with ICU serum compared with ward serum or no serum. Statistical significance was determined using the non-parametric Kruskal–Wallis test, followed by Dunn’s *post hoc* test. ***P < 0.001. Data were obtained from *n* = 4 independent experiments.

To test this, we established an *in vitro* endothelial complement opsonization model (Fig. 4c). Endothelial cells were incubated overnight with pooled serum from ICU patients, ward patients, or control medium (no serum). After washing, fresh complement-active serum from pooled healthy donors (NHS) was added, and complement deposition was assessed by flow cytometry. To account for complement potentially carried over from the pre-incubation serum, parallel conditions received heat-inactivated NHS after identical pre-incubation and washing. In these conditions, C3c and C5b-9 signals were low throughout; the small signal detected after pre-incubation with ICU serum was lower to the baseline deposition on untreated cells exposed to fresh NHS. IgM and IgG levels did not differ after serum pre-incubation, arguing against a contribution of (auto)reactive antibodies (online supplementary material, Fig. S2). Endothelial cells pre-treated with ICU serum showed significantly increased C3c opsonization and C5b-9 deposition compared with ward serum or control medium (Fig. 4d and e, both *P* < 0.001). These results indicate that exposure to serum from patients with COVID-19 admitted to the ICU renders endothelial cells more susceptible to complement opsonization.

### Soluble complement inhibitors in long COVID

Although levels of soluble complement inhibitors generally normalized over time, a subset of individuals showed persistently elevated factor H at 6 weeks, prompting the hypothesis that sustained elevation may predispose to long COVID.

To test this, serum samples from individuals with long COVID (*n* = 79), post-COVID-19 controls without fatigue (*n* = 18), and pre-pandemic healthy controls (*n* = 47) were analyzed (Table 2). C1-INH and factor I levels were comparable across all three groups (Fig. 5a and d). Compared with pre-pandemic controls, individuals with long COVID had higher C4BP (Fig. 5b, *P* = 0.02) and factor H levels (Fig. 5c, *P* < 0.001). No differences were observed between individuals with long COVID and post-COVID-19 controls without fatigue, suggesting that these elevations do not solely reflect current symptom burden. Of note, factor H was the only inhibitor for which levels were significantly higher in no-fatigue controls than in healthy controls.

**Figure 5:**
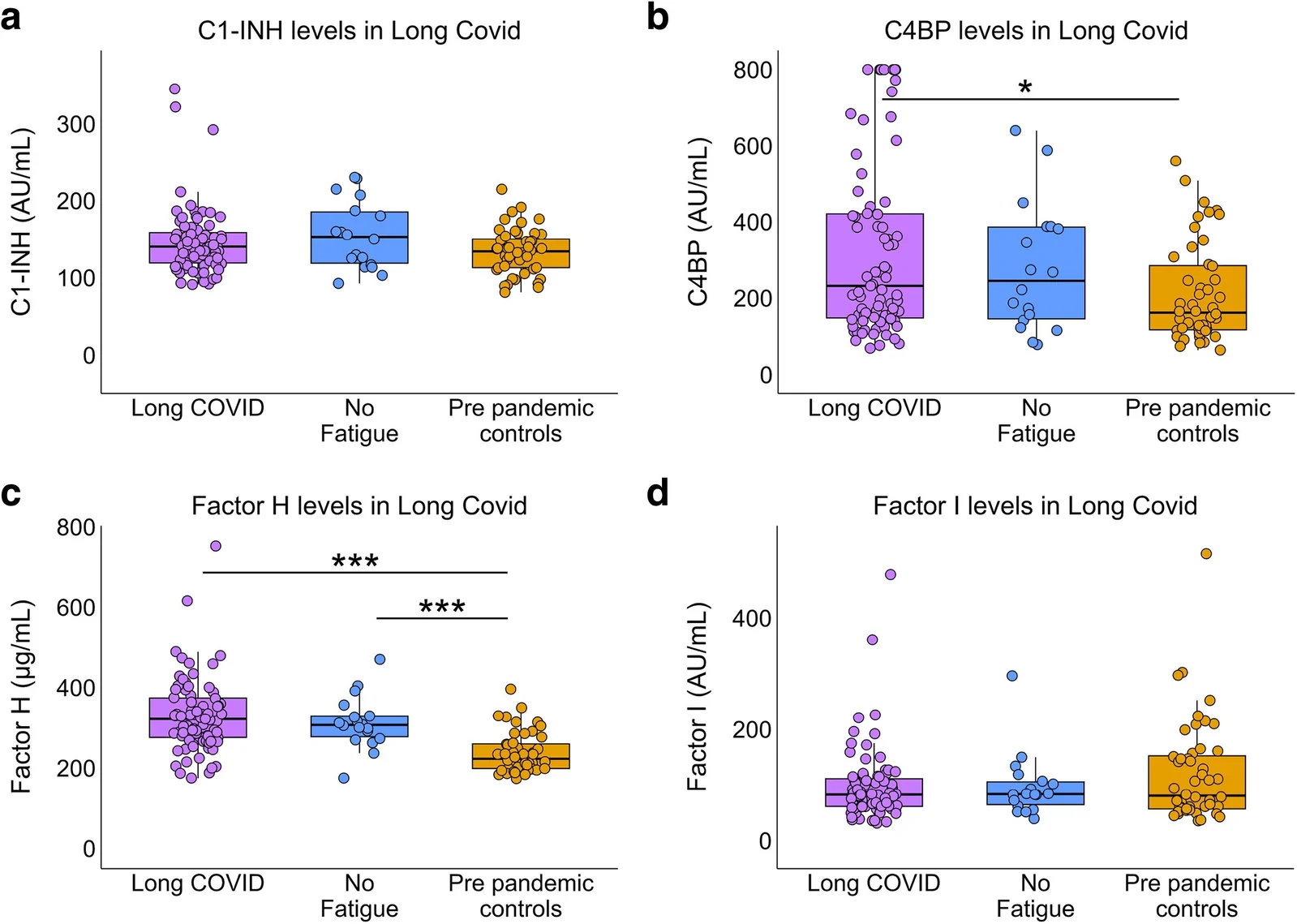
Soluble complement inhibitors in long COVID. (a) Serum levels of C1-INH among long COVID patients, no-fatigue post-COVID controls, and pre-pandemic healthy controls are similar. (b) Long COVID patients exhibited higher C4BP (*P* = 0.02) than pre-pandemic controls; differences versus non-fatigue controls were not significant. (c) Serum levels of factor H were elevated in both long COVID patients and no-fatigue post-COVID controls compared with pre-pandemic controls (*P* < 0.001). (d) No differences in the levels of factor I between the different groups. Statistical significance for multi-group comparisons was assessed using the non-parametric Kruskal–Wallis test, followed by Dunn’s *post hoc* test. *P < 0.05; **P < 0.01; ***P < 0.001.

**Table 2:** Demographics long COVID.

	Long COVID (*n* = 79)	No-fatigue post-COVID (*n* = 18)	Pre-pandemic healthy controls (*n* = 47)
Male	25 (32%)	3 (17%)	31 (66%)
Age in years ± SD	48 ± 12	36 ± 10	51 ± 8
Time since infection, days	342 (262–476)	140 (100–208)	
Fatigue score	62 (55–69)	9 (4–12)	

## Discussion

In this longitudinal study of patients with severe COVID-19, we demonstrate systemic complement activation, particularly in patients admitted to the ICU, which correlated with disease severity and the need for mechanical ventilation, but not with in-hospital mortality. Soluble complement inhibitors were increased rather than reduced, with factor H positively correlating with sC5b-9. Functionally, serum from patients admitted to the ICU increased endothelial susceptibility to complement deposition. In individuals with long COVID, factor H and C4BP levels remained elevated compared with pre-pandemic controls.

Complement activation has consistently been linked to severe COVID-19, with sC5b-9 being associated with respiratory failure and adverse outcomes [6, 10, 34, 35]. Our findings that sC5b-9 tracks with disease severity and mechanical ventilation are therefore in line with previous reports. While complement activation contributes to pathogen clearance, excessive activation can exacerbate tissue injury. This has been demonstrated in SARS-CoV-1 models, where C3 deficiency reduced lung pathology and improved respiratory function [36]. Additionally, abrogation of C5b-9 formation due to C7 deficiency attenuated disease [37]. In line with our own findings, recent work has shown that C5b-9 deposition increases on primary lung endothelial cells upon incubation with COVID-19 patient serum [38], supporting a model in which patient serum factors enhance cellular susceptibility to complement attack.

Despite pronounced complement activation, we observed increased levels of soluble inhibitors. C1-INH was elevated in both patients admitted to the ICU and to the ward compared with healthy controls. This aligns with earlier cross-sectional reports describing increased C1-INH levels in COVID-19, with higher levels in patients with more severe disease [39, 40]. C4BP levels were likewise increased, consistent with earlier observations [41]. Our longitudinal data additionally revealed distinct kinetics between groups: although C4BP levels at admission were similar in ICU patients, ward patients, and healthy controls, levels rose selectively in ICU patients during the course of disease before declining again, whereas ward patient levels remained comparable to controls throughout.

A particularly striking finding was the elevation of factor H and its positive correlation with sC5b-9. Although counterintuitive, increased factor H levels have been reported in other COVID-19 cohorts. In contrast, proteomic studies have suggested that factor H-related proteins, rather than factor H itself, track with disease severity [42]. In chronic kidney disease, altered factor H-to-factor H-related protein ratios, together with glycocalyx damage, are associated with increased complement activation [43]. As our ELISA specifically detects factor H and not related proteins, our data suggest that although factor H is abundant in circulation, it may fail to exert adequate regulatory activity at the cell surface. Potential mechanisms include competition between SARS-CoV-2 and factor H for heparan sulfate binding [44], glycocalyx disruption preventing surface binding [45] or interference by viral proteins such as ORF8, which can bind C3b and thereby block factor H-mediated regulation [46]. Factor I levels were likewise elevated, consistent with proteomic and ELISA-based studies [41, 47]. An alternative explanation for altered inhibitor levels or function would be the presence of autoantibodies against complement regulators, as described in other inflammatory conditions [20, 48]. However, IgG reactivity against factor H, factor I, C4BP, and C1-INH was infrequent and comparably distributed between ICU and ward patients, arguing against an autoantibody-driven mechanism.

We did not quantify the native complement components C3, C4, and C5, but the reported changes in these components run opposite to, rather than parallel with, those of the inhibitors. A meta-analysis of 19 studies comprising 3764 patients found both C3 and C4 to be significantly lower in patients with severe disease or non-survivor status, consistent with consumption [49], a pattern reproduced in cohorts stratified by respiratory support and by survival [50]. A biphasic course has also been described, with C3 and C4 rising in mild disease before falling in severe disease, indicating that the measured concentration reflects the net of hepatic acute-phase synthesis and consumption and is therefore sensitive to the timing of sampling. Native C5 is rarely quantified; the terminal pathway is instead assessed through C5a and sC5b-9, both consistently elevated in severe disease [6, 10], as in our cohort.

Collectively, our data suggest that during severe COVID-19, serum-derived factors modify endothelial surfaces, disrupting the normally low-avidity interaction of complement inhibitors with the cell surface [31, 33]. In contrast to conventional complement-regulator deficiencies, in which the inhibitors themselves are absent or dysfunctional, we propose that the soluble inhibitors in COVID-19 remain intrinsically competent; the dysregulation originates not from the inhibitors but from infection-induced perturbation of their engagement with the cell surface. This results in elevated circulating inhibitor levels but insufficient local protection, allowing complement-mediated cellular injury and coagulopathy. This situation phenotypically resembles conditions of factor H deficiency or functional inhibition, such as atypical hemolytic uremic syndrome, which are characterized by pro-coagulant states [48], although the underlying mechanism differs fundamentally. Therapeutic strategies may therefore need to focus not on increasing inhibitor concentrations, but on restoring their localization to the cell surface. Bispecific antibodies that recruit circulating factor H to cellular membranes have shown promise in experimental models [51, 52], and may represent a strategy to correct defective local complement regulation.

Complement activation has also been implicated in long COVID [53]. In our cohort, group-level differences between individuals with long COVID and no-fatigue post-COVID controls were limited, although C4BP was elevated compared with pre-pandemic controls. Factor H levels were increased in both post-COVID groups. Notably, a subset of individuals with long COVID exhibited markedly elevated inhibitor levels, suggesting heterogeneity and potential immunopathological subgroups rather than a uniform phenotype.

Several limitations should be acknowledged. First, the immunological context in which the complement system was activated in this cohort reflects a naïve response to a novel viral pathogen. While this enhances the value of our findings in understanding primary immune responses, it may limit their generalizability to the current epidemiological context, in which most individuals have been vaccinated, previously infected, or exposed to different viral variants. Second, a pre-analytical consideration is our use of serum rather than EDTA-plasma for complement measurements. Although EDTA-plasma halts ex vivo complement activation at the moment of collection, Conformité Européenne—*in vitro* diagnostic-certified sC5b-9 immunoassays such as the Quidel MicroVue sC5b-9 Plus assay validate serum as an acceptable matrix, provided that reference ranges are interpreted accordingly. Because all samples in our cohort were collected and processed identically, and because inter-plate assay performance was consistent across experiments, any contribution of matrix choice to baseline sC5b-9 levels applies equally across groups. Between-group comparisons and longitudinal trends therefore reflect genuine biological differences rather than differential pre-analytical activation. Reassuringly, the magnitude and kinetics of sC5b-9 elevation observed here are consistent with those reported in studies using EDTA-plasma [6, 10], supporting the biological validity of our findings. Third, a major limitation is the lack of direct assessment of factor H binding or function at the cell surface. We attempted to address this using the endothelial model, but the avidity of factor H was too low to allow reliable detection by flow cytometry. Alternative approaches, such as imaging or proximity-based assays, may be required in future studies to confirm whether factor H engagement at the cell surface is impaired. Finally, our study focused exclusively on patients with COVID-19 and did not include comparator groups with other respiratory viral infections, such as influenza, or critically ill non-COVID ICU patients. Elevated fluid-phase complement activation markers have been described across diverse critical illnesses, and we therefore cannot formally exclude that part of the observed activation reflects critical illness or ARDS *per se* rather than SARS-CoV-2-specific pathophysiology. However, our data robustly demonstrate pronounced complement activation in severe COVID-19, and earlier studies directly comparing COVID-19 with influenza and non-COVID acute respiratory failure reported higher sC5b-9 levels in COVID-19 [6], indicating that complement activation is a prominent feature of severe SARS-CoV-2 infection, even if shared with other forms of ARDS.

## Conclusion

In summary, severe COVID-19 is characterized by robust complement activation despite elevated levels of soluble inhibitors. This paradox suggests that the inhibitors themselves are not deficient; rather, complement dysregulation likely reflects impaired engagement of these inhibitors with the cell surface, compounded by overwhelming systemic activation. The increased susceptibility of endothelial cells to complement deposition supports this model. Consequently, therapeutic strategies aimed at restoring inhibitor function at the cell surface may prove more effective than systemic complement modulation alone.

## Supplementary Material

uxag064_Supplementary_Data

## Data Availability

The data supporting the findings of this study are available from the corresponding author, Leendert Trouw (l.a.trouw@lumc.nl), on reasonable request.

## Clinical trial registration

International Clinical Trials Registry Platform, NL8589.

## Abbreviations:

ABTS: 2,2′-azino-bis(3-ethylbenzothiazoline-6-sulfonic acid)
aHUS: atypical hemolytic uremic syndrome
AIDS: acquired immunodeficiency syndrome
ARDS: acute respiratory distress syndrome
AU: arbitrary units
BMI: body mass index
BSA: bovine serum albumin
C1-INH: C1-inhibitor
C4BP: C4b-binding protein
CE-IVD: Conformité Européenne—*in vitro* diagnostic
CI: confidence interval
COVID-19: coronavirus disease 2019
DVT: deep vein thrombosis
EDTA: ethylenediaminetetraacetic acid
EGM-2: endothelial cell growth medium 2
FCS: fetal calf serum
FH: factor H
FI: factor I
Hi-NHS: heat-inactivated normal human serum
HIV: human immunodeficiency virus
HUVEC: human umbilical vein endothelial cell
ICU: intensive care unit
IgG: immunoglobulin G
IgM: immunoglobulin M
IQR: interquartile range
LLOQ: lower limit of quantification
MASP: mannose-binding lectin-associated serine protease
NHS: normal human serum
OR: odds ratio
PBS: phosphate-buffered saline
PCR: polymerase chain reaction
PE: pulmonary embolism
SARS-CoV-2: severe acute respiratory syndrome coronavirus 2
sC5b-9: soluble C5b-9 (soluble membrane attack complex)
SST: serum separator tube
VWF: von Willebrand factor
