# Supplementary material for: Dysregulated complement activation in COVID-19: the role of soluble complement inhibitors
Source: Clin Exp Immunol. 2026 Oct 16;220(1):uxag064. doi: 10.1093/cei/uxag064 (PMC13741236; doi:10.1093/cei/uxag064)
Supplement: Supplementary file 1 [file uxag064_supplementary_data.docx]

**Supplementary materials**

**Dysregulated complement activation in COVID-19: The Role of Soluble Complement Inhibitors**

**Authors:**

Stef van der Meulen^1^, Haiyu Wang^1^, Saskia Nugteren^1^, Frederique Ponchel^2-3^, Anna H. E. Roukens^4^, Cilia R. Pothast^5^, Mirjam H.M. Heemskerk^5^, Kyra A. Gelderman^6^, Jeroen C. J. Eikenboom^7-8^, Bernard M. van den Berg^8^, Wendy Stam^9^, Coert Margadant^9^, Simone A. Joosten^4^, on behalf of the BEAT-COVID study group, Leendert A. Trouw^1^

**Affiliations:**

^1^ Department of Immunology, Leiden University Medical Center, Leiden, The Netherlands.

^2^ Leeds Institute of Rheumatic and Musculoskeletal Medicine, University of Leeds, Leeds, UK

^3^ Département de Rhumatologie, Université Jean Monnet Saint-Etienne, CHU Saint-Etienne, INSERM, Mines Saint-Etienne, SAINBIOSE U1059, Saint-Etienne, France.

^4^ Leiden University Center for Infectious Diseases (LUCID), Leiden University Medical Center, Leiden, The Netherlands.

^5^ Department of Hematology, Leiden University Medical Center, Leiden, The Netherlands.

^6^ Laboratory Medical Immunology, Department of Immunology, Erasmus Medical Center, Rotterdam, The Netherlands

^7^ Department of Internal Medicine, Division of Thrombosis and Hemostasis, Leiden University Medical Center, Leiden, The Netherlands.

^8^ The Einthoven Laboratory for Vascular and Regenerative Medicine, Dept of Internal Medicine, Nephrology, Leiden University Medical Center, Leiden, The Netherlands

^9^ Institute of Biology, Gorlaeus Laboratory, Leiden University, Leiden, The Netherlands

**Corresponding author:**

Leendert A. Trouw, PhD

Department of Immunology, L3-33A

Leiden University Medical Center

Postbus 9600, 2300 RC Leiden

The Netherlands

Tel 0031-715263869

l.a.trouw@lumc.nl

**Supplementary methods**

**BEAT-Covid Cohort**

The BEAT-COVID cohort was collected at the Leiden University Medical Centre (LUMC), Leiden, The Netherlands. Individuals diagnosed with PCR-positive COVID-19, who required hospital admission between April 2020 and March 2021 were invited to participate.

Clinical care was provided according to local and national guidelines and not influenced by study participation The initial patient care (wave-1, March 2020–August 2020) was supportive care only. The patient care which was provided later on (wave-2, August 2020–March 2021) included the administration of dexamethasone. Since our study was initiated shortly after the onset of the pandemic in The Netherlands, criteria for hospital admission were also different between the waves. In wave-1, hospital admission was limited to the more severe cases, and generally admission occurred later after onset of symptoms compared to wave-2 as a result of capacity and national guidelines. Lastly, SARS-CoV-2 PCR testing was only available for hospital-admitted patients whereas it was mandatory for all individuals with symptoms in the 2nd wave; hence, people were aware of the infection earlier and may thus have behaved differently.

Patients were included both from the regular ward as well as from the ICU. Inclusion criteria were: admission at LUMC, SARS-CoV-2 PCR-test positive, and minimally 18 years old. Exclusion criteria were: no informed consent from the patient or a representative. All participants were unvaccinated against SARS-CoV-2. Upon signing informed consent, blood samples were collected 3 times a week (Monday–Wednesday–Friday) during hospitalization and at an outpatient visit 6–12 weeks post discharge. Statistical sample size calculation was not performed, the sample size was determined based on availability.

In addition to the patients with COVID-19 disease, a control group of age and sex matched healthy individuals was assembled in July 2020 for comparative analyses. None of these healthy volunteers had experienced the infection yet, as was confirmed by serological analyses. Semi-quantitative detection of SARS-CoV-2 anti-nucleocapsid (N) protein IgG antibodies was performed on the Abbott Architect platform[1, 2]. In this antibody chemiluminescent microparticle immunoassay (CMIA) test, the SARS-CoV-2 antigen-coated paramagnetic microparticles bind to the IgG, respectively, IgM antibodies that attach to the viral nucleocapsid protein in human serum samples. The Sample/Calibrator index values of chemiluminescence in relative light units (RLU) of 1.40 (IgG assay) respectively 1.00 (IgM assay) and above were considered as positive per the manufacturer’s instructions. Samples were collected from 8 males and 4 females (as the ratio of hospital admissions at that time was 2:1), with a median age of 64 (range 60–72). Healthy volunteers were sampled 5 times, also at 2 day intervals and samples were processed identically to those from the individuals included in the BEAT-COVID cohort.

**SCODA-severity-scores**

The Severity of Coronavirus Disease Assessment (SCODA) disease severity-score was developed to track day-to-day disease severity in hospital admitted COVID-19 patients[3]. The score was based on the 4C mortality score, but constant parameters were substituted with parameters associated with breathing and oxygenation to better reflect the daily condition and its changes[3]. The SCODA score was developed such that there was continuous scoring during admission on the ward and ICU to permit longitudinal assessment of severity during both types of admission. The SCODA score consisted of the following parameters: respiratory rate, peripheral oxygen saturation on room air (ward only), P/F Ratio (ICU only), oxygen flow (ward only), FiO2 (ICU only), Glasgow Coma Scale (GCS), blood urea level and C-reactive protein (CRP)[3]. The most severely ill patients admitted to the ICU reached a maximum severity-score of 17.

We defined the recovery time point, per definition after the highest severity-score for that individual patient, as a SCODA score of a daily severity-score of ≤ 7, with no subsequent increases. SCODA scores were included in the analysis when matched serum samples and measurements on the soluble analytes were available.

**Supplementary data**

Since autoantibodies against complement regulators have been described in inflammatory conditions and could affect inhibitor levels or function, we screened all available samples for IgG reactivity against Factor H, Factor I, C4BP and C1-INH by ELISA, using BSA as a control antigen. Patients with BSA reactivity were excluded, leaving 33 ICU patients, 38 ward patients and 12 healthy controls for analysis. Autoantibody positivity was infrequent in all groups; anti-FH (Figure 1A, ICU 2/33, ward 4/38, healthy 0/12), anti-C4BP (Figure 1B, 6/33, 0/38, 0/12), anti-FI (Figure 1C, 1/33, 0/38, 0/12) and anti-C1INH (Figure 1D, 3/33, 2/38, 1/12). Fourteen patients were positive for at least one inhibitor, with a comparable overall frequency in ICU and ward patients (7/33 vs 6/38, p = 0.76, Fisher's exact test). These data indicate that the elevated concentrations of soluble complement inhibitors observed in COVID-19 are unlikely to be driven by an autoantibody response.

**Supplementary Figure 1. Autoantibodies against soluble complement inhibitors in ICU patients, ward patients and healthy controls.**

IgG reactivity against complement regulators was measured by ELISA in all available samples of the BEAT-COVID cohort, using BSA as a control antigen. Samples with reactivity against BSA were considered non-specific and excluded, leaving 33 ICU patients, 38 ward patients and 12 healthy controls for analysis. Each point on the x-axis represents an individual patient; corresponding coordinates on the y-axis indicate the values of all samples collected over time from that patient. Levels are expressed in arbitrary units per mL (AU/mL). **(A)** Anti-Factor H reactivity; positivity was detected in 2/33 ICU patients, 4/38 ward patients and 0/12 healthy controls. **(B)** Anti-C4b-binding protein reactivity; 6/33 ICU patients, 0/38 ward patients and 0/12 healthy controls. **(C)** Anti-Factor I reactivity; 1/33 ICU patients, 0/38 ward patients and 0/12 healthy controls. **(D)** Anti-C1-inhibitor reactivity; 3/33 ICU patients, 2/38 ward patients and 1/12 healthy controls.

**IgM and IgG deposition on endothelial cells**

To exclude that the complement activation observed on endothelial cells was mediated by IgM or IgG antibodies present in the pooled patient sera, we performed a single validation experiment in which endothelial cells were incubated overnight with pooled ICU serum, pooled ward serum, or a no-serum control, and subsequently stained for surface-bound IgM and IgG by flow cytometry. Comparable levels of IgM and IgG deposition were observed across all conditions, making an antibody-driven mechanism an unlikely explanation for the enhanced complement deposition induced by ICU serum.

**Supplementary Figure 2. IgM and IgG deposition on endothelial cells by patient serum**

IgM and IgG staining by flow cytometry after overnight incubation of endothelial cells with ICU serum, ward serum, or no-serum control showed no differences between groups, excluding IgM- or IgG-mediated complement activation.

**References**

1.	Escribano P, Alvarez-Uria A, Alonso R, Catalan P, Alcala L, Munoz P, Guinea J. Detection of SARS-CoV-2 antibodies is insufficient for the diagnosis of active or cured COVID-19*.* Sci Rep 2020; **10**:19893.

2.	Maine GN, Lao KM, Krishnan SM, Afolayan-Oloye O, Fatemi S, Kumar S, VanHorn L, Hurand A, Sykes E, Sun Q. Longitudinal characterization of the IgM and IgG humoral response in symptomatic COVID-19 patients using the Abbott Architect*.* J Clin Virol 2020; **133**:104663.

3.	Joosten SA, Smeets MJR, Arbous MS, Mannien J, Laverman S, Driessen MMG, Cannegieter SC, Roukens AHE, Leiden University Medical Center B-Cg. Daily disease severity in patients with COVID-19 admitted to the hospital: The SCODA (severity of coronavirus disease assessment) score*.* PLoS One 2023; **18**:e0291212.
